# A twin transition in regulatory toxicology: moving toward Chemicals 2.0 and phasing out animal testing

**DOI:** 10.1093/toxsci/kfae130

**Published:** 2024-10-03

**Authors:** Andrew P Worth, Elisabet Berggren

**Affiliations:** European Commission, Joint Research Centre (JRC), Ispra, Italy; European Commission, Joint Research Centre (JRC), Ispra, Italy

**Keywords:** chemicals policy, chemical safety assessment, animal testing, non-animal methods, risk management, roadmap

## Abstract

The European regulatory framework on chemicals is at a crossroads. There are calls for the framework to be more effective, by better protecting people and the environment. There is also room for it to be more efficient and cost-effective, by harmonizing assessment practices across sectors and avoiding the need for unnecessary testing. At the same time, there is a political commitment to phase out animal testing in chemical safety assessments. In this commentary, we argue that these needs are not at odds with each other. On the contrary, the European Commission’s roadmap to phase out animal testing could also be the transition pathway to a more efficient, effective, and sustainable regulatory ecosystem. Central to our proposal is a framework based on biological reasoning in which biological questions can be answered by a choice of methods, with non-animal methods progressively becoming the only choice. Within this framework, a tiered approach to testing and assessment allows for greater efficiency and effectiveness, while also introducing considerations of proportionality and cost-effectiveness. Testing strategies, and their component methods, should be developed in tandem and judged in terms of their outcomes, and the protection levels they inform, rather than their ability to predict the outputs of animal tests.

In the current EU regulatory framework for assessing the safe use of chemicals, there is room for improved testing strategies that go beyond current information requirements and maximize protection of the human health and the environment. This would also be a desirable outcome in the context of the European Commission’s roadmap towards phasing out animal testing for chemical safety assessment (European Commission 2023, 2024).

New approach methodologies (NAMs), based on scientific understanding and supported by innovative technologies, have developed rapidly in recent years. Although these methods are usually not based on animal testing, here we use the term non-animal methods, to avoid ambiguity. The major challenge is no longer the lack of methods to replace animal testing but how to make them practically available and acceptable to regulators. There is a need to create a regulatory framework that builds on the experience obtained since the 1960s (when the first European legislation on chemicals entered into force) but is also able to keep pace with scientific progress in methodology development. To this end, three major objectives should be: (i) enabling a minimal level of assessment for all chemicals on the market to more effectively protect human health and the environment, (ii) harmonizing information and testing requirements across the chemicals framework to gain efficiencies, and (iii) introducing a tiered approach to testing to include proportionality and allow for cost-benefit optimization. When conceiving of a regulatory framework to meet these objectives, we base our thinking on the simplified classification system outlined in our vision for Chemicals 2.0 (Berggren and Worth 2023).

However, we recognize that there is little support from many stakeholders to modernize the system for assessing chemicals. A recent report on so-called sociotechnical barriers (Čavoški A et al. 2024) identified that the major issues are a lack of common understanding and trust between stakeholders. There is a reluctance to abandon entrenched assessment practices and there is uncertainty (even anxiety) about transitioning to a new paradigm. This creates a vicious cycle in which the challenge is simplified and presented as a scientific and technological one, leading regulators to demand the development and validation of accurate methods at a faster pace, to which the scientific community responds by trying harder and harder to meet increasing expectations. Related to this, there have been calls for urgent mobilization of national and regional resources for the demonstration of reproducibility and reliability of methods developed in single laboratories (OECD 2023a). Additional funding is of course welcome but it needs to be targeted in a strategic way, and this includes a serious discussion about the way we validate methods in the context of Integrated Approaches to Testing and Assessment (IATA).

In this commentary, we first outline how the transition to a non-animal based system can be based on multiple pathways to acceptance, each with a different level of ambition. We then look at the current legal framework and see how it could embrace non-animal methods by revising the current hazard assessment criteria. Finally, we suggest that information and testing requirements could be partially harmonized across sectors (Oltmanns et al. 2023) by introducing a tiered testing strategy which would also allow for a more effective and efficient protection. The transition to Chemicals 2.0 reflects the interrelated ambitions to embrace the principle of “one substance, one assessment”, support the concept of safe and sustainable by design, and phase out animal testing in chemical safety assessments.

## The transition to animal-free safety assessment: multiple pathways of acceptance

The introduction of non-animal methods into regulatory practice faces a number of barriers, especially the sociotechnical ones mentioned above. The need to gain confidence is widely acknowledged, as is the desire to avoid setting up new business barriers. However, it takes time for innovations, such as non-animal methods, to diffuse through the technology adoption life cycle. An obvious way of overcoming a barrier is to break it down into smaller steps. With this in mind, we think it is helpful to distinguish between five pathways to acceptance, each differing in terms of the decision-making context and associated level of ambition. These are not intended to be mutually exclusive. Indeed, the transition will depend on pursuing all pathways at the same time, making the “best choice” of pathway on a case-by-case basis.

### Augmenting

At the lowest level (smallest barrier), under the current legislation, non-animal methods can be introduced to provide information on a concern that would otherwise be neglected, in cases where no information is explicitly required or there are no animal methods to address the specific concern. An example of this is the use of the Threshold of Toxicological Concern (TTC) approach for low-level exposures (Patlewicz et al. 2022). Augmenting can be regarded as the “better than nothing” approach.

### Complementing

At the next level is the use of non-animal methods to enhance confidence in the outcome of an already-required animal test. This increases confidence in current protection measures, by strengthening the weight of evidence underpinning the decisions.

### Repurposing

At the third level, an already established non-animal method, usually with high confidence, can be repurposed and then employed to fulfil information requirements for a different concern or context of use. In current legislation, this might be when a test method used under one piece of legislation is transferred to fulfil an information requirement in another.

### Replacing

When a non-animal method or testing strategy is considered to fulfil the same information requirements as an animal study, the latter can then be omitted. This is the ultimate goal under current legislation, and for local health effects this has worked out well, but as we try to address more complex concerns such as chronic environmental effects and systemic health effects, non-animal methods do not provide the same kind of information and are thus not accepted as replacements.

### Rebuilding

Because the prospects for replacement under current legislation are limited, and arguably, we have reached that limit, it is timely to start redesigning the regulatory framework to be fit for non-animal methods. This is the fifth level, representing the largest barrier and the ultimate ambition.

## Current legal framework

The United Nations Globally Harmonized System for classification of dangerous chemicals, GHS (UN 2023a), is implemented in the EU through the CLP Regulation (EU 2008). The GHS has as far as feasible common classification criteria with the Transport of Dangerous Goods, TDG (UN, 2023b). Therefore, when changing the scope of current GHS classes in the EU classification system, the revisions must be agreed by the UN ECETOC committee, overseeing the work of the GHS and TDG subcommittees. There is, however, strong support to revise the GHS classification system by introducing non-animal methods, and in 2015, an informal working group on non-animal testing methods was established. The working group has since revised the chapters on skin corrosion/skin irritation, severe eye damage/eye irritation and skin sensitization to include non-animal testing and non-testing methods that fulfil the current criteria for classification.

The challenge we now face with systemic human health and chronic environmental effects is that the current GHS classes are not suitable for non-animal methods that do not allow prediction of the adverse effects observed in animal studies or in humans. In addition, there is the problem of the large overlap of systemic classifications, i.e., a chemical classified in one of the current classes is often additionally classified in one or more others. We have investigated the overlap of harmonized classified chemicals within the EU as listed in Annex VI of the CLP Regulation (see Table 1). These overlaps can also be thought of as conditional probabilities. For example, if a chemical is classified as a mutagen, there is a 90% probability it is also a carcinogen (a class which includes mutagenic and non-mutagenic carcinogens). Conversely, if a chemical has been classified as a carcinogen, there is a 45% probability it is also a mutagen.

**Table 1.** Overlap of harmonized classified chemicals within the EU as listed in Annex VI of the CLP Regulation, as updated with the 18th Adaptation to Technical Progress (EU, 2022).

	Total number of chemicals classified in this class	Chemicals only in this class among the classes relevant to systemic toxicity	Chemicals classified at least in one other class relevant to systemic toxicity	Overlap of chemicals classified specified by class					
				Mutagenicity	Carcinogenicity	Reproductive toxicity	STOT-RE	STOT-SE	Acute toxicity
Mutagenicity	575	18 (3%)	557 (97%)	–	517 (90%)	76 (13%)	88 (15%)	18 (3%)	118 (21%)
Carcinogenicity	1151	387 (34%)	764 (66%)	517 (45%)	–	121 (11%)	171 (15%)	58 (5%)	264 (23%)
Reproductive toxicity	388	96 (25%)	292 (75%)	76 (20%)	121 (31%)	–	163 (42%)	33 (9%)	199 (51%)
STOT-RE	552	74 (13%)	478 (87%)	88 (16%)	171 (31%)	163 (30%)	–	43 (8%)	372 (67%)
STOT-SE	297	113 (38%)	184 (62%)	18 (6%)	58 (20%)	33 (11%)	43 (14%)	–	161 (54%)
Acute toxicity	1703	985 (58%)	718 (42%)	118 (7%)	264 16%)	199 (12%)	372 (22%)	161 (9%)	–

Abbreviations: STOT-RE, specific target organ toxicity (repeated exposure); STOT-SE, specific target organ toxicity (single exposure).

The other overlaps are less predictable but are likely based on the commonality of underlying mechanisms. Accordingly, we suggest that the classification system could be made more efficient by relying on non-animal approaches providing evidence of such mechanisms. For context, it should be noted that the harmonized classifications are made by expert judgment using multiple lines of evidence that meet the classification criteria. This started in 1967 with the first EU legal act on classification and labeling, Directive 67/548/EEC, later replaced with the CLP Regulation based on the GHS. During this timeframe, the classification criteria have themselves been revised several times. We believe these classifications are protective, but we need to recognize that they were based on different kinds of evidence and that expert judgements also altered during this long period. A classification system could equally be based on evidence from non-animal methods, including scope for expert judgment. The latter point is often overlooked but could be an important way of gaining experience while exploring defined approaches (standardized algorithms for interpreting data).

The CLP Regulation is a horizontal piece of legislation within the EU, and all chemicals are subject to hazard classification regardless of marketed volume or use. The regulation does not contain any information requirements, but rather criteria indicating which types of data can be used for hazard assessment. The REACH regulation (EU 2006) is also horizontal, and applicable to chemicals manufactured in or imported to the EU in large volumes. The information requirements are set up to fulfill the CLP criteria. Other pieces of legislation, instead aim at the authorization of certain uses, e.g., plant protection products, biocides, pharmaceuticals. The information requirements under these legal acts also enable hazard classification under the CLP Regulation. There is a vast amount of “downstream” EU legislation related to protection of workers, consumers and the environment based on the CLP classifications.

It is important that legal requirements are proportionate to provide the right level of protection, without creating business barriers and losing the many benefits from chemicals used in our daily lives. It is therefore necessary to weigh the costs and benefits underpinning safe and sustainable progress. The efficiency of the current system has been called into question as we have only managed to assess a minority of the chemicals on the market since 1967. Nevertheless, we believe the most hazardous ones are covered, as they were classified based on concerns raised by Member States, in order to protect, e.g., workers, consumers or the environment. This means that existing classifications can be used as a means of calibrating a new classification system to ensure the same level of protection, before applying the new classification system to the vast majority of chemicals that have not been assessed (Berggren and Worth 2023).

## Future legal framework

Classification criteria based on methods predicting intermediate effects could indirectly address several adverse outcomes, e.g., endocrine disruption can lead to both carcinogenicity and reproductive toxicity in humans. Based on this reasoning, establishing a reduced number of non-overlapping classes would reduce the burden of proof. The reduction of classes would avoid overlaps between the GHS classes while also covering concerns that in the current system based on downstream adverse effects in animals might be missed (either because there is no animal method available or because the animal method is insufficiently sensitive).

Key to embracing change is the recognition that the current system is not perfect but at the same time has resulted in a valuable body of knowledge on chemical properties and effects. No revision to the GHS should require that already assessed chemicals need to be re-classified unless additional effects are introduced into the system. A new classification system can be phased into the current one by letting the two systems coexist based on the requirement that they provide the same level of protection. In this hybrid system, the same labeling elements would be applied for corresponding concerns. If the new NAM-based classification system reaches this level and is accepted as an alternative, one of the two classifications can be chosen when implementing the GHS based on the building block principle (UN 2023a). Consistent with the building block approach, countries are free to determine which of the GHS building blocks are applied in different parts of their systems, as long as they do so consistently.

However, the risk of building further on the current system is that we will continue to add to the evidence burden. Some non-animal methods might only be accepted as complementary methods, providing further confidence in the animal data without ever fulfilling replacement (according to a reviewer, developmental immunotoxicity might be such a case). Another scenario is that some non-animal methods are only accepted as partial replacements. For example, an in vitro battery for Developmental Neurotoxicity (OECD 2023b) could be included as partial evidence for a current class (Reproductive toxicity with adverse effects on development of the offspring) and lead to classification, but it would not cover other adverse outcomes leading to classification for the same endpoint. In other words, we might create a system that becomes too complex for its purpose. To avoid this, we should not aim for perfection. We do not need to know everything about chemicals. Rather, we need to set up a system that effectively and efficiently protects human health and the environment from chemicals of concern while guaranteeing that beneficial chemicals are available.

Before proposing a new classification system, we need to create the necessary knowledge to support such an alternative. To this end, the EPAA designation for systemic toxicity was launched in 2023 (EPAA 2023) with the aim of providing a safe co-creation space for experts to develop and evaluate the feasibility of such a system.

## A cross-sector tiered approach to testing and assessment

**Fig. 1.**
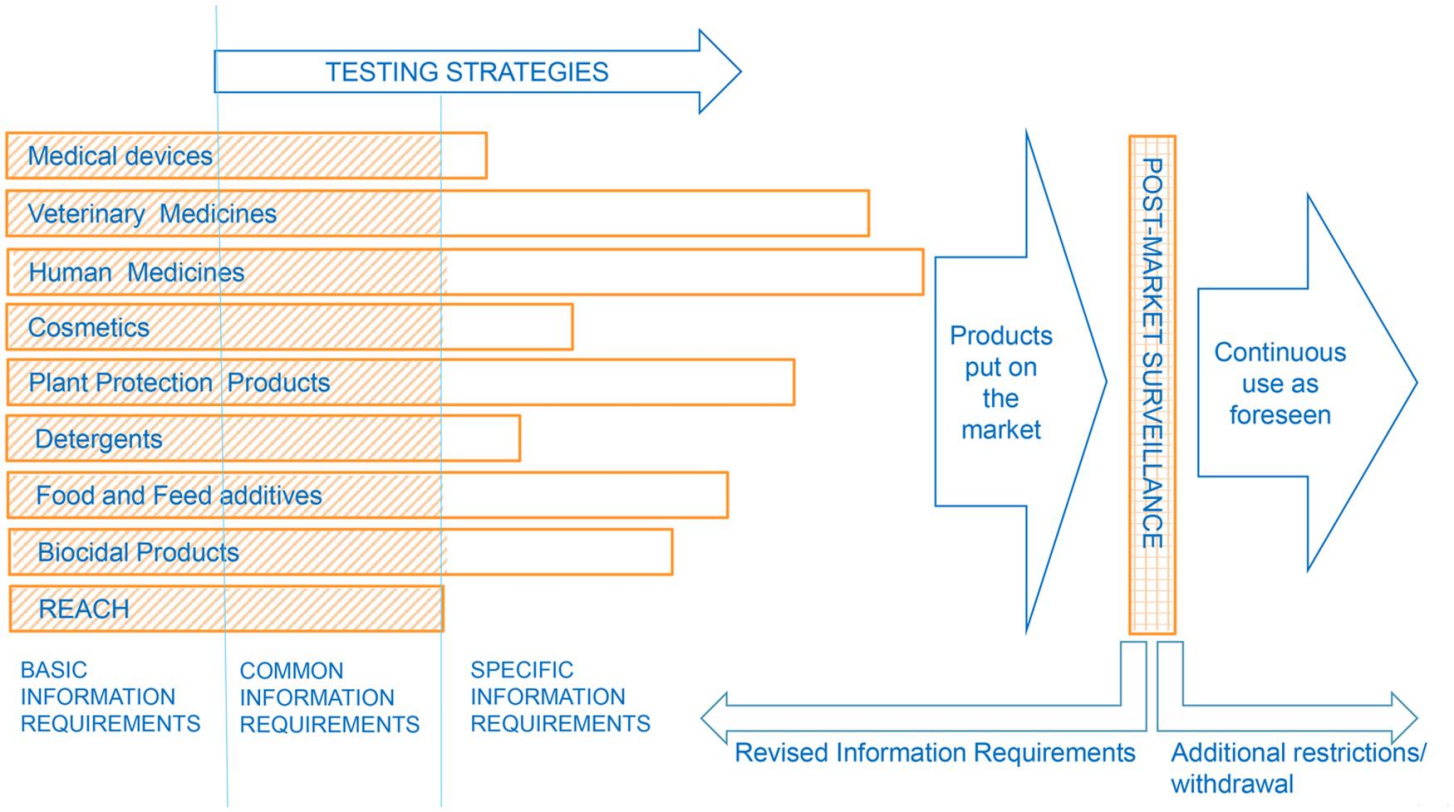
The bars illustrate information requirements under different pieces of EU legislation and their arbitrary lengths indicate that the information required in a specific area is more or less extensive. The shading of the bars indicates information requirements that are common to different pieces of legislation. Basic information requirements are a part of the common information requirements.

To support a more effective and efficient safety assessment for all chemicals, and to introduce proportionality considerations, we suggest introducing three levels of information (Fig. 1). The information is obtained by applying a tiered approach, in which each level builds on previous levels. Each level can include its own testing strategies, to reach the protection goals of specific pieces of legislation.

### BASIC information requirements

In principle, these would be applicable to the entire chemical universe comprising an estimated 100,000 chemicals.

At this level, the information gathered includes existing data as well as data generated by computational modeling. The TTC approach (Patlewicz et al. 2022) would also apply at this level. The objective is to identify NEGATIVES (“green chemicals”) with high confidence, so they can be used in the context of Safe and Sustainable by Design (SSbD) (European Commission 2022). Negatives at this level will not necessarily cover all concerns, but will provide a high level of confidence for low concern. In addition, this level could be reinforced with standardized high throughput testing enabling an Early Warning System to identify potential risks and consequently identify chemicals to be further tested at the next level. Other chemicals will be considered of low concern, with potential use in products that are safe and sustainable by design.

### COMMON information requirements

Basic hazard and exposure information applicable to chemicals registered/authorized under REACH and vertical (sectoral) pieces of legislation, enabling generic risk assessments. Estimated 20,000 chemicals.

At this level in vitro testing and invertebrate testing (in case the full step to non-animal testing is not considered feasible to meet the protection goal; PrecisionTox Consortium 2023) are introduced, including highly standardized methods for hazard classification. The objective is to identify HIGH CONCERN POSITIVES (“red chemicals”) with high confidence, so they can be risk managed appropriately by authorities.

### SPECIFIC information requirements

Additional hazard and exposure information for chemicals with specific uses, enabling detailed risk assessments. The scope of such requirements is roughly estimated to be around 10,000 chemicals across the chemicals acquis in addition to around 30,000 cosmetics ingredients with existing data (Cosmetics Europe 2023).

This level is directly related to specific legal acts and introduces more bespoke non-animal (or in specific cases, invertebrate) testing for detailed risk assessments. The objective is to identify LOW CONCERN POSITIVES (“orange chemicals”) with high confidence, so that safe use can be demonstrated by companies. At this level, chemicals subject to a specific piece of legislation, identified as green at the first level, could be further tested to ensure their safe use. In addition, chemicals that were identified as red, could be further tested, in case no replacements can be identified, to ensure as safe use as possible, considering social-economic interests.

This is where Next Generation Risk Assessment and probabilistic risk assessment come in Maertens et al. (2024). Assessments would follow a biological questions-based approach with multiple assessment pathways possible (Berridge et al. 2024). This is consistent with the ASPIS Safety Profiling Algorithm being developed by the ASPIS consortium. In this approach, there should be no need to assess every endpoint, in view of mechanistic overlaps (common upstream key events in Adverse Outcome Pathways), and it would be possible to adopt a conservative/precautionary approach to risk management where uncertainties are large or unknown. There will also be different levels of accuracy in toxicity and exposure assessment depending on the final use. For example, human and veterinary medicines require greater accuracy to determine the therapeutic index.

We also suggest to introduce a safety net into the system, where not already applied, namely the use of post-market environmental monitoring (Finckh et al. 2024) and biomonitoring to identify unaddressed risks (Reale et al. 2024). In such studies, chemicals with high levels of occurrence and which may be of concern are identified. This information could then trigger further safety assessment and risk management, and could also be used as an alert to monitor structurally/biologically similar compounds. This monitoring information could be regarded as an Early Warning System Alert at the lowest level, with already assessed chemicals being monitored for a better-informed safety assessment.

## Discussion

In the recent scientific literature, there is growing interest and speculation about the future of the EU chemicals regulatory system (e.g., Botham et al. 2023; Brescia et al. 2023; Alexander-White 2024; Godderis et al. 2024). The aim of this commentary is to contribute to the ongoing debate, building on our previous vision for Chemicals 2.0 (Berggren and Worth, 2023). There are multiple pressures for change, and a multitude of possible transition pathways, making this a complex political problem. We have focused on the role of non-animal methods in this transition, which we consider to be central.

In agreement with recent studies (Čavoški et al. 2024), we also believe the sociocultural barriers to be greater than the scientific and technical ones. Of course, there will always be gaps in our knowledge and assessment methodology (e.g., ECHA 2023), because research never ends. The question though is how to apply the best available technologies today, and what lines of new research genuinely promise to add value in the context of regulatory decision-making. Sadly, a lot of current research does not translate into useful assessment practices. Another important consideration is that after 30 years of incremental progress in introducing non-animal methods into regulation, we have now reached the limit of replacement potential. We therefore need a step change to a system where decisions are reached on the basis of new kinds of evidence (particularly from non-animal methods but also monitoring data). In other words, we need to redesign the system. However, this appears to be a herculean task, given that the current practices are deeply entrenched.

As a workaround, we are advocating for a hybrid system—a period of co-evolution. Our proposal is that the tiered testing strategy and the post-market surveillance could be implemented already in the short-medium term, and built on top of the current system. This implies a willingness from the beginning to accept some information requirements based on animal testing when considered unavoidable (acknowledging that there will always be a spectrum of opinions on what is avoidable or not). However, the animal tests should eventually be displaced when there is sufficient confidence in the use of non-animal methods. This is where the Commission’s roadmap to phase out animal testing comes in European Commission (2024).

Information requirements for the complex endpoints will continue to be addressed by using IATA. This view is neither controversial nor new, with proposals for “integrated testing strategies” dating back to the preparations for REACH (Grindon et al. 2006). It is imperative though that these IATA are designed and evaluated in an appropriate way from the outset. We argue that IATA, and their component methods, should not be judged in terms of their predictivity of current animal studies, because this not only prolongs our entrenched reliance on animal testing but also limits our ability to extend the scope of protection (to concerns not yet covered). Instead, IATA should be judged on how they could be used to ensure an equivalent or better level of protection. We therefore need to find a way of articulating these protection goals.

To ensure that IATA are future-proof against technological developments, they should be designed according to the information they generate and the risk management outcomes they inform. We therefore advocate for a plug-and-play approach to building IATA. An IATA starts as a generic framework in which new methods can be “pluggedin,” provided they meet certain specifications. This is consistent with the notion of a biological-based questions approach, as proposed by Berridge and co-workers (Berridge et al. 2024), in that the information IATA generate should respond to a set of biological questions, including questions we have yet to formulate. In other words, the IATA and their component methods should be developed in parallel, bearing in mind the five pathways to acceptance described above. This also has important implications for how an eventual European Test Method and Validation Strategy should be implemented, following a proposal by the Dutch and German Competent Authorities (CARACAL 2024).

In summary, we believe that the transition to Chemicals 2.0 and the phasing out of animal testing are aligned and mutually reinforcing in terms of their goals. Central to the transition, we propose a biological-based questions approach to the development of IATA in which non-animal methods are progressively plugged in while animal tests are switched out. There is a need for consensus on the philosophy underlying the design, evaluation, and application of these IATA. Most research endeavors today are chasing rainbows, seeking to optimize the ability to predict of animal data. This is a flawed approach which is itself becoming a barrier to progress. As the German-American evolutionary biologist, Ernst Mayer, once said, “Scientific progress consists in the development of new concepts, which often requires the abandonment of old ones.”

## Disclaimer

This article reflects the views of the authors and does not necessarily reflect those of the European Commission.
